# Epidermal Rac1 regulates the DNA damage response and protects from UV-light-induced keratinocyte apoptosis and skin carcinogenesis

**DOI:** 10.1038/cddis.2017.63

**Published:** 2017-03-09

**Authors:** Jayesh Deshmukh, Ruth Pofahl, Ingo Haase

**Affiliations:** 1 Department of Dermatology, University of Cologne, Kerpener Strasse 62, Cologne 50937, Germany

## Abstract

Non-melanoma skin cancer (NMSC) is the most common type of cancer. Increased expression and activity of Rac1, a small Rho GTPase, has been shown previously in NMSC and other human cancers; suggesting that Rac1 may function as an oncogene in skin. DMBA/TPA skin carcinogenesis studies in mice have shown that Rac1 is required for chemically induced skin papilloma formation. However, UVB radiation by the sun, which causes DNA damage, is the most relevant cause for NMSC. A potential role of Rac1 in UV-light-induced skin carcinogenesis has not been investigated so far. To investigate this, we irradiated mice with epidermal Rac1 deficiency (Rac1-EKO) and their controls using a well-established protocol for long-term UV-irradiation. Most of the Rac1-EKO mice developed severe skin erosions upon long-term UV-irradiation, unlike their controls. These skin erosions in Rac1-EKO mice healed subsequently. Surprisingly, we observed development of squamous cell carcinomas (SCCs) within the UV-irradiation fields. This shows that the presence of Rac1 in the epidermis protects from UV-light-induced skin carcinogenesis. Short-term UV-irradiation experiments revealed increased UV-light-induced apoptosis of Rac1-deficient epidermal keratinocytes *in vitro* as well as *in vivo*. Further investigations using cyclobutane pyrimidine dimer photolyase transgenic mice revealed that the observed increase in UV-light-induced keratinocyte apoptosis in Rac1-EKO mice is DNA damage dependent and correlates with caspase-8 activation. Furthermore, Rac1-deficient keratinocytes showed reduced levels of p53, *γ*-H2AX and p-Chk1 suggesting an attenuated DNA damage response upon UV-irradiation. Taken together, our data provide direct evidence for a protective role of Rac1 in UV-light-induced skin carcinogenesis and keratinocyte apoptosis probably through regulating mechanisms of the DNA damage response and repair pathways.

Non-melanoma skin cancers (NMSC) are the most common skin cancers.^1, 2, 3^ Although their etiology is multifactorial, UVB irradiation by the sun is the most relevant cause for NMSC.^4^ The UVB part of solar UV radiation is directly absorbed by DNA. UV-light-induced DNA damage typically leads to the formation of two major DNA lesions: cyclobutane pyrimidine dimers (CPDs) and 6-4 pyrimidine photoproducts (6-4 PPs).^5, 6, 7^ CPDs form the majority of the DNA mutations which occur as a result of UV-light-induced DNA damage.^8, 9^

In many plants and animals, but not in placental mammals, CPDs are efficiently repaired and removed by specialized enzymes: CPD photolyases (CPDPL).^10^ After activation by visible light, photolyases directly and lesion specifically reverse UV-light-induced DNA lesions.^10, 11^ In placental mammals, removal of CPDs is primarily carried out by a highly conserved and complex multistep mechanism called nucleotide excision repair (NER). Loss of function mutations in the pathway regulating NER have been shown to result in dramatically increased skin carcinogenesis in Xeroderma pigmentosum patients.^12^

To protect from skin carcinogenesis keratinocytes exhibit a preventive mechanism called DNA damage response (DDR), which can have different physiological outcomes such as cell cycle arrest, activation of the DNA repair machinery, apoptosis or senescence; depending upon the level of DNA damage and the ability of keratinocytes to repair the damage.^13, 14^ The ATR-Chk1 pathway is primarily activated in UV-light-induced DDR.^15^ Cells with severe DNA damage are partially eliminated by apoptosis. It is believed that, if this mechanism of elimination fails, some cells with severe DNA damage accumulate mutations, resulting in malignant transformation.^7, 13^

UV-light-induced keratinocyte apoptosis results in the formation of sunburn cells in the epidermis.^16, 17^ Sunburn cells are apoptotic keratinocytes possessing severely and irreversibly damaged DNA as a result of UV radiation. These sunburn cells are destined to death and exhibit characteristic morphological features: a dark pyknotic nucleus and scanty, eosinophilic cytoplasm.^16, 17^ UV-light-induced keratinocyte apoptosis is thought to involve the intrinsic and the extrinsic pro apoptotic signaling pathways, depending on the activation of caspases-9 and -8, respectively. In the intrinsic pathway, activation of caspase-9 is dependent on DNA damage and up-regulation of p53. The extrinsic apoptotic pathway can be initiated by activation of various cell surface death receptors such as Fas, TNFR-1 and TRAIL-R2,^18, 19^ which then trigger the activation of caspase-8.

Rac1 is a small Rho family GTPase of the Ras superfamily. In epidermal keratinocytes, the Rac1 activator Tiam1 has been shown to prevent keratinocyte apoptosis after growth factor starvation and heat shock treatment.^20^ In several experimental settings and in different cell types, Rac1 has been shown to have an important role in either pro-apoptotic or anti-apoptotic mechanisms.^21, 22, 23, 24^ Inhibition or deletion of Rac1 in HeLa cells and pancreatic cancer cells attenuated the DDR upon UV-irradiation or ionizing radiation, whereas the DDR was enhanced in a hepatic tumor model.^25, 26, 27^

Rac1 is over-expressed in various types of human cancer, such as testicular, gastric and breast cancer as well as oral squamous cell carcinomas (SCCs).^28^ Over-activity of Rac1 was also observed in various human SCCs and in cell lines derived from head and neck SCCs.^29, 30^ Mice with targeted deletion of the Rac1 guanine nucleotide exchange factor (GEF) Tiam1, show decreased skin papilloma formation in a multistep chemical carcinogenesis model, but also a relative increase in SCCs, as compared with benign skin papillomas.^31^ Targeted deletion of Rac1 has been shown to impair skin papilloma formation in a chemically induced skin carcinogenesis model in mice in a mixed 129 Sv/ C57BL/6 background. Although loss of Rac1 from epidermal keratinocytes decreased epidermal hyper-proliferation, keratinocyte apoptosis was not affected.^32^ Inhibition of Rac1 activity by a pharmacological inhibitor, NSC23766, in epidermal keratinocytes also reduced skin papilloma formation in a chemical skin carcinogenesis model.^29^ We have shown recently that Rac1 activity is, on one hand, required for the development of skin papillomas in human papilloma virus type 8 transgenic mice. On the other, absence of Rac1 from epidermal keratinocytes facilitated progression of these papillomas to SCCs.^33^

Given the proposed role of Rac1 in skin tumor formation and the known importance of UV-light as the major carcinogen in skin, we asked whether UV-light-induced skin carcinogenesis is influenced by Rac1.

## Results

### Skin erosions upon long-term UV-irradiation in mice with epidermis-specific deletion of Rac1

**Figure 1:**
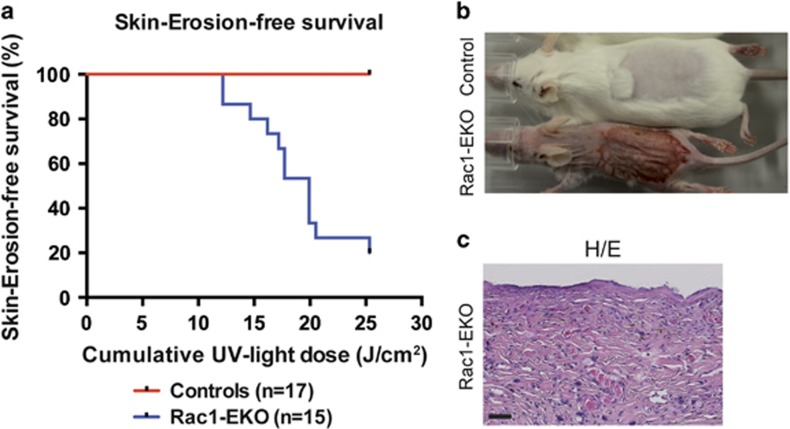
Epidermis-specific deletion of Rac1 leads to severe skin erosions after chronic-UV-irradiation. (**a**) Kaplan–Meier curve of skin erosion-free survival of controls and Rac1-EKO upon chronic-UV-irradiation. (**b**) Representative photographs of control and Rac1-EKO mice treated with chronic-UV-irradiation at a cumulative dose of 24.12 J/cm^2^. (**c**) H/E staining of a skin section from the erosive area of the Rac1-EKO mouse shown in (**b**). Scale bar=100 *μ*m

To investigate whether epidermis-specific deletion of Rac1 leads to altered UV-light-induced skin carcinogenesis, we irradiated wild-type mice (controls) and epidermis-specific Rac1-deficient mice^34, 35, 36^ (Rac1-EKO) with increasing doses of UV-light according to a protocol used previously.^33, 37^ None of the 17 control mice developed any visible skin changes up to a cumulative dose of 25 J/cm^2^, whereas 12 out of 15 (80%) Rac1-EKO mice developed areas of denuded, erosive skin within the irradiation field, starting from 12 J/cm^2^ (Figures 1a and b). These lesions first appeared as erythema with scaling. Later we observed erosions covered with crusts, which enlarged in size and covered the whole irradiation field. Histological analysis revealed that the areas of erosive skin were devoid of epidermis (Figure 1c).

### Epidermis-specific deletion of Rac1 increases UV-light-induced keratinocyte apoptosis *in vivo* and *in vitro*

Our studies had shown that mice with epidermis-specific Rac1 deficiency develop severe skin erosions upon long-term UV-irradiation (Figures 1a–c). We, therefore, asked whether apoptosis was altered in Rac1-EKO mice upon UV-irradiation. To investigate this, we carried out short-term UV irradiation experiments with a single dose of 1 J/cm^2^ UVB.

**Figure 2:**
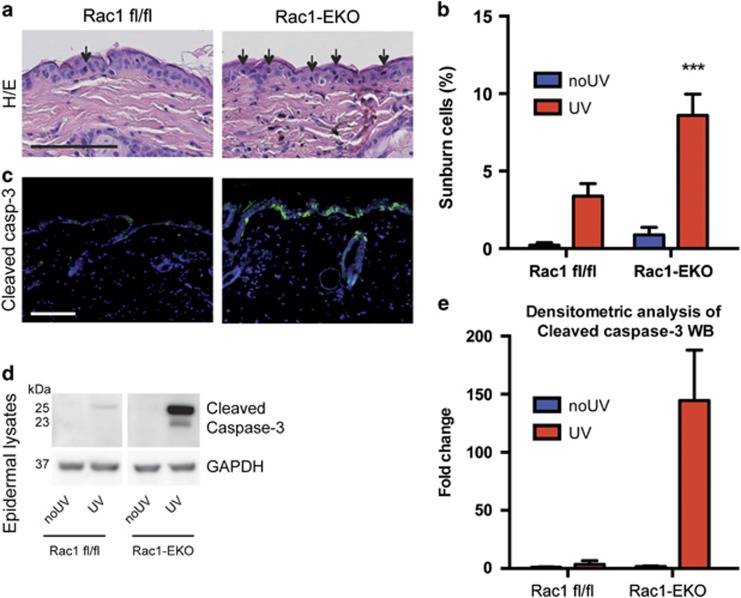
Epidermis-specific deletion of Rac1 increases UV-light-induced keratinocyte apoptosis *in vivo*. (**a**) H/E staining of UV-irradiated skin of Rac1 fl/fl and Rac1-EKO mice at 12 h after UV-irradiation. Black arrows indicate sunburn cells. (**b**) Graph shows the percentage number of sunburn cells at 12 h with (red bars) or without (blue bars) UV-irradiation in Rac1 fl/fl (*n*=4) and Rac1-EKO (*n*=5) mice. The percentage of sunburn cells within the epidermis after UV-irradiation in Rac1 fl/fl mice was 3.4%, whereas in Rac1-EKO mice it was 8.6% of total epidermal keratinocytes. Non-irradiated samples showed <1% sunburn cells in both the genotypes. (**c**) Immunostainings against cleaved caspase-3 (green) of UV-irradiated skin of Rac1 fl/fl and Rac1-EKO mice at 12 h after UV-irradiation. Nuclei are stained in blue. Scale bar=100 *μ*m. (**d**) Western blot analysis of cleaved caspase-3 from epidermal lysates of untreated (no UV) and UV-light treated (UV) Rac1 fl/fl and Rac1-EKO mice. Non-irradiated controls showed no bands for cleaved capsase-3, whereas samples of irradiated epidermis showed cleaved caspase-3-specific bands at ~25 kDa and 23 kDa. Numbers on the left denote molecular weights in kDa. (**e**) Graph shows densitometry analysis of western blot in (**d**). Error bars show S.D. Asterisks show *P*-value<0.001

At 12 h after UV-irradiation, histological analysis of skin sections revealed a significant increase (*P*-value<0.001) in the number of sunburn cells (apoptotic cells as a result of UV-irradiation) in Rac1-EKO mice as compared with Rac1 fl/fl mice (Figures 2a and b). In agreement with the H/E stainings, immunostainings showed a clear increase in the number of cleaved caspase-3-positive cells in Rac1-EKO epidermis (Figure 2c). This was further confirmed by western blot analysis of epidermal lysates (Figure 2d). In line with our immunostaining results, we observed much stronger bands for cleaved caspase-3 in Rac1-EKO mice than in Rac1 fl/fl mice upon UV-irradiation (Figure 2d). Densitometry analysis revealed a striking increase in cleaved caspase-3 levels in the epidermis of Rac1-EKO mice compared with Rac1 fl/fl mice (Figure 2e).

**Figure 3:**
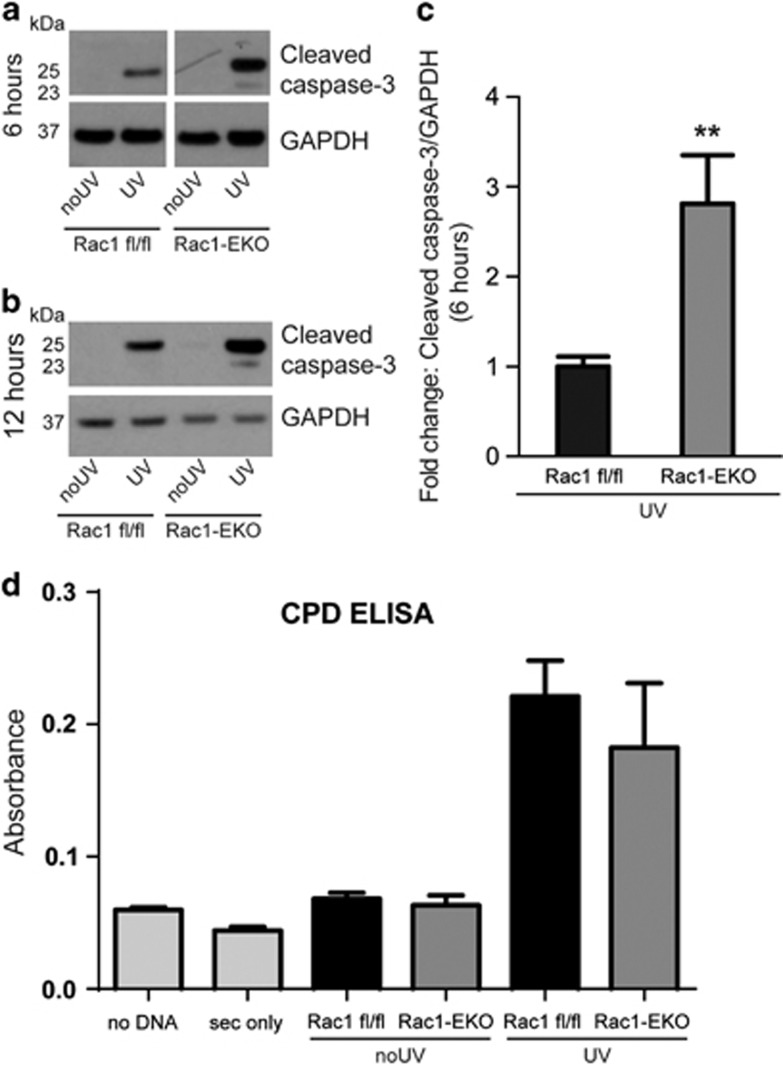
Rac1 deficiency increases sensitivity towards UV-light-induced keratinocyte apoptosis *in vitro*. (**a** and **b**) Western blot analysis of cleaved caspase-3 from Rac1 fl/fl and Rac1-EKO cultured keratinocytes at 6 h (**a**) and at 12 h (**b**) with (UV) or without (no UV) UV-irradiation. (**c**) Densitometry analysis of cleaved caspase-3 at 6 h after UV-irradiation from Rac1 fl/fl and Rac1-EKO keratinocytes. (**d**) Quantification of CPDs by CPD ELISA carried out from genomic DNA isolated from Rac1 fl/fl and Rac1-EKO mouse primary keratinocytes immediately after UV-irradiation. Error bars show S.D. Asterisks show *P*-value<0.01

To investigate whether Rac1-deficient keratinocytes in culture (Supplementary Figure S2) also exhibit a similar response to UV-light, we irradiated Rac1 fl/fl and Rac1-EKO keratinocytes with 0.5 J/cm^2^ of UV-light. We observed stronger bands for cleaved caspase-3 in lysates of UV-irradiated Rac1-EKO keratinocytes as compared with lysates of Rac1 fl/fl keratinocytes at 6 h and 12 h after UV-irradiation (Figures 3a–c). To exclude a difference between Rac1 fl/fl and Rac1-EKO keratinocytes in the extent of UV-light-induced immediate DNA damage we analyzed the amount of CPDs immediately after UV-irradiation. Both Rac1-EKO and control cells showed comparable levels of CPDs (Figure 3d).

These data show that Rac1 deficiency increases keratinocyte apoptosis upon UV-irradiation *in vivo* as well as *in vitro* suggesting that cell autonomous mechanisms are responsible for the increased susceptibility towards UV-light-induced apoptosis.

### DNA damage has an important role in increased UV-light-induced keratinocyte apoptosis in Rac1-EKO epidermis *in vivo*

**Figure 4:**
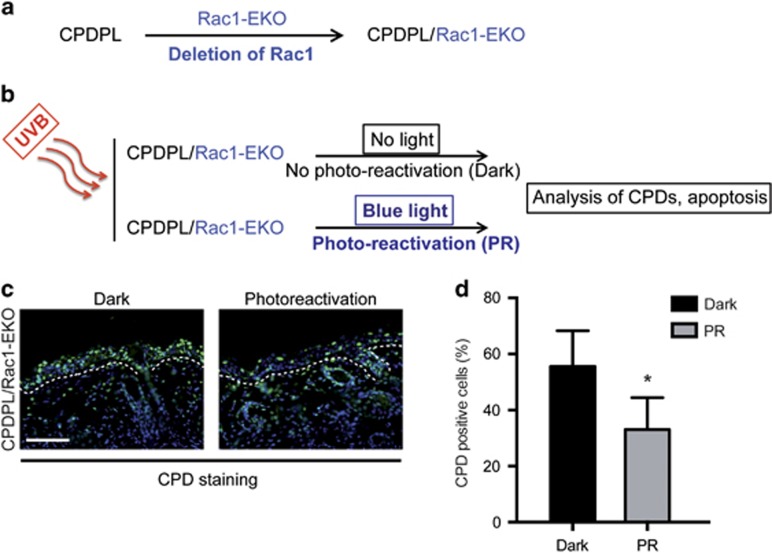
Generation of CPDPL/Rac1-EKO mice and photoreactivation of CPDPL in CPDPL/Rac1-EKO mice. (**a** and **b**) Schematic representation of generation of CPDPL/Rac1-EKO mice and the photoreactivation experiments, respectively. (**c**) Immunostainings against CPD (green) of UV-irradiated skin of CPDPL/Rac1-EKO mice kept in the dark and under the photoreactivation lamp. Nuclei are stained in blue. Dashed line demarcates border between epidermis and dermis. Scale bar=100 *μ*m. (**d**) Graph shows quantification of CPD-positive cells from UV-irradiated CPDPL/Rac1-EKO mice kept in the dark and under the photoreactivation (PR) lamp (*n*=5 each). Error bars show S.D. Asterisk show *P*-value<0.05

Previously, it has been shown that the repair of CPDs by enzymatic photo-reactivation through CPDPL, expressed as a transgene under the control of the keratin 14 (K14) promoter, rescues UV-light-induced apoptosis in these transgenic mice.^38^ To investigate whether forced repair of CPDs would rescue increased UV-light-induced apoptosis in Rac1-EKO mice, we generated CPDPL/Rac1-EKO mice by breeding CPDPL transgenic mice^38^ with Rac1-EKO mice (Figure 4a). We then irradiated CPDPL/Rac1-EKO mice with a single dose of 1 J/cm^2^ UV-light and kept one group in the dark (Dark) and the other under blue light for photo-reactivation (PR) for 12 h. Analysis of CPDs and apoptosis was carried out on histological sections of skin tissue samples taken 12 h after UV-irradiation (Figure 4b). Immunostaining against CPDs showed a significant reduction (*P*-value=0.018) in the number of CPD-positive cells in the PR group (33%) as compared with the Dark group (56%) (Figures 4c and d), suggesting partial repair of CPDs in the PR group.

**Figure 5:**
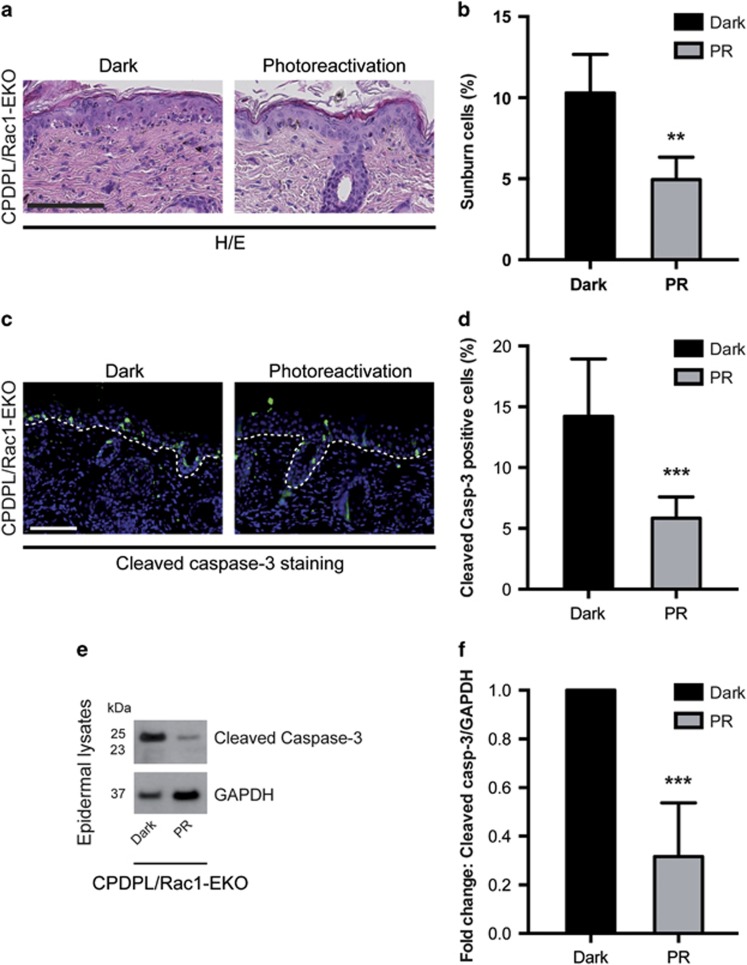
DNA damage has an important role in increased UV-light-induced keratinocyte apoptosis in Rac1-EKO epidermis *in vivo*. (**a** and **c**) H/E stainings and immunostainings against cleaved caspase-3 (green) of UV-irradiated skin of CPDPL/Rac1-EKO mice kept in the dark and under the photoreactivation lamp. Nuclei are stained in blue. Dashed line in **c** demarcates border between epidermis and dermis. Scale bar=100 *μ*m. (**e**) Western blot analysis of cleaved caspase-3 from epidermal lysates of UV-irradiated CPDPL/Rac1-EKO mice kept in the dark and under the photoreactivation lamp. GAPDH was used as a loading control. Numbers on the left denote molecular weight in kDa. (**b**,**d**, and **f**) Graphs show quantification of sunburn cells (*n*=5, 4) (**b**), cleaved caspase-3 positive cells (*n*=9, 8) (**d**) and densitometric analysis of cleaved caspase-3 western blots (*n*=4, 6) (**f**) from UV-irradiated CPDPL/Rac1-EKO mice kept in the dark (black bars) and under the photoreactivation (PR) lamp (gray bars). Error bars show S.D. Asterisks show *P*-value. **<0.01, ***<0.001

Moreover, histological analysis revealed a significant reduction (*P*-value=0.0045) in the number of sunburn cells in the PR group (4.9%) as compared with the Dark group (10.3%) (Figures 5a and b); this was confirmed for cleaved caspase-3-positive cells (5.8% *versus* 14.2% *P*-value=0.0005) (Figures 5c and d). In agreement with this, western blot analysis of epidermal lysates for cleaved caspase-3 showed strong bands in the Dark group and much weaker bands in PR group (Figures 5e and f). These data show that forced repair of CPDs by photo-reactivation through CPDPL significantly reduces UV-light-induced keratinocyte apoptosis in Rac1-EKO mice.

### Rac1-deficient keratinocytes show an altered DDR upon UV-irradiation *in vitro*

**Figure 6:**
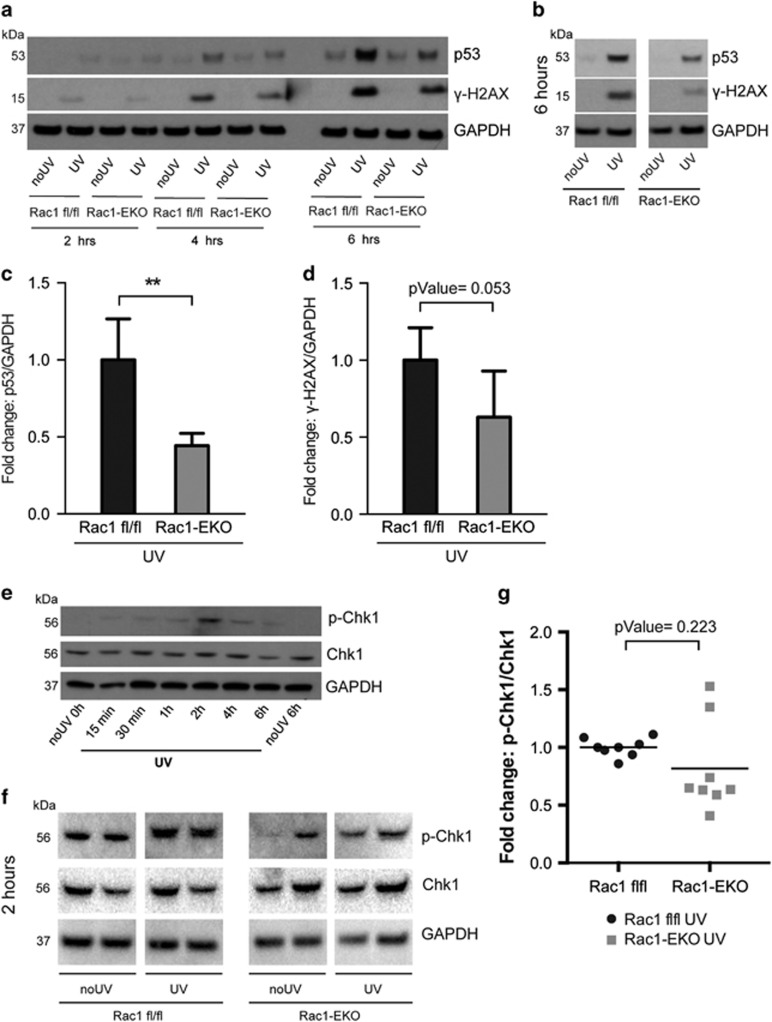
Rac1-deficient keratinocytes show an altered DNA damage response upon UV-irradiation. (**a**) Western blot analysis of p53 and phosphorylation of H2AX (*γ*-H2AX) from Rac1 fl/fl and Rac1-EKO keratinocytes at indicated time points with (UV) or without (no UV) UV-irradiation. Time-course experiments revealed highest levels of p53 and *γ*-H2AX at 6 h after UV-irradiation in Rac1 fl/fl keratinocytes as compared with non-irradiated controls. Therefore, we used the 6 h time point for further analysis of p53 protein levels and *γ*-H2AX. (**b**) Representative western blot analysis of p53 and *γ*-H2AX from Rac1 fl/fl and Rac1-EKO cultured keratinocytes at 6 h with (UV) or without (no UV) UV-irradiation. (**c** and **d**) Densitometry analyses of fold change of p53 (*n*=3 each) (**c**) and *γ*-H2AX (*n*=5 each) (**d**) normalized to GAPDH in Rac1 fl/fl and Rac1-EKO samples at 6 h after UV-irradiation. Error bars show S.D. ***represent *P*-value<0.001. (**e**) Western blot analysis of p-Chk1 and Chk1 from Rac1 fl/fl keratinocytes at indicated time points after UV-irradiation. Time-course experiments revealed maximum phosphorylation of Chk1 at 2 h after UV-irradiation in Rac1 fl/fl primary keratinocytes. Therefore, in the following experiments, phosphorylation of Chk1 was investigated at 2 h after UV-irradiation. (**f**) Representative western blots of p-Chk1, Chk1 from Rac1 fl/fl and Rac1-EKO cultured keratinocytes at 2 h with (UV) or without (no UV) UV-irradiation. GAPDH was used as loading control. Numbers on the left denote molecular weights in kDa. (**g**) Dot plot showing densitometry analysis results of fold change of p-Chk1 normalized to total Chk1 in Rac1 fl/fl and Rac1-EKO samples after UV-irradiation

Our *in vivo* results suggest that increased keratinocyte apoptosis in Rac1-deficient epidermis is DNA damage dependent. To investigate a potential role of Rac1 in the UV-light-induced DDR pathway, we analyzed protein levels of p53 and phosphorylation of the histone H2A.X variant (*γ*-H2AX). p53 protein levels and *γ*-H2AX are known to be regulated by the ATR/Chk1 DDR pathway.^13, 15^ *γ*-H2AX is a widely used marker of DDR. Western blot analysis showed very low levels of p53 and no *γ*-H2AX in non-irradiated samples (Figures 6a and b). Rac1-EKO samples showed significantly reduced (*P*-value=0.002) levels of p53 as compared with Rac1 fl/fl samples after UV-irradiation (Figures 6b and c). Similar to p53, levels of *γ*-H2AX were reduced (*P*-value=0.053) in Rac1-EKO samples as compared with Rac1 fl/fl samples after UV-irradiation (Figures 6b and d).

Next, we analyzed phosphorylation of Chk1 as a readout for the activity of the ATR-Chk1 pathway, which is primarily activated in UV-light-induced DDR.^15^ Western blot analysis showed very weak or no bands in non-irradiated controls (Figures 6e and f). At 2 h after UV-irradiation, much weaker bands for p-Chk1 were observed in Rac1-EKO keratinocytes compared with Rac1 fl/fl keratinocytes (Figure 6f). Densitometry analysis indeed revealed reduced phosphorylation of Chk1 in Rac1-EKO keratinocytes in a series of eight samples each, suggesting reduced activation of the ATR/Chk1 DDR pathway in Rac1-EKO keratinocytes after UV-irradiation (Figure 6g). Despite high variability in the levels of p-Chk1 phosphorylation most of the Rac1-EKO samples showed reduced phosphorylation as compared with the controls. These data show that Rac1 deficiency in primary mouse keratinocytes inhibits signaling via the ATR/Chk1 DDR pathway upon UV-irradiation, suggesting an important role of Rac1 in the DDR pathway in the epidermis.

### The increase in UV-light-induced apoptosis in Rac1-deficient keratinocytes requires activation of caspase-8

**Figure 7:**
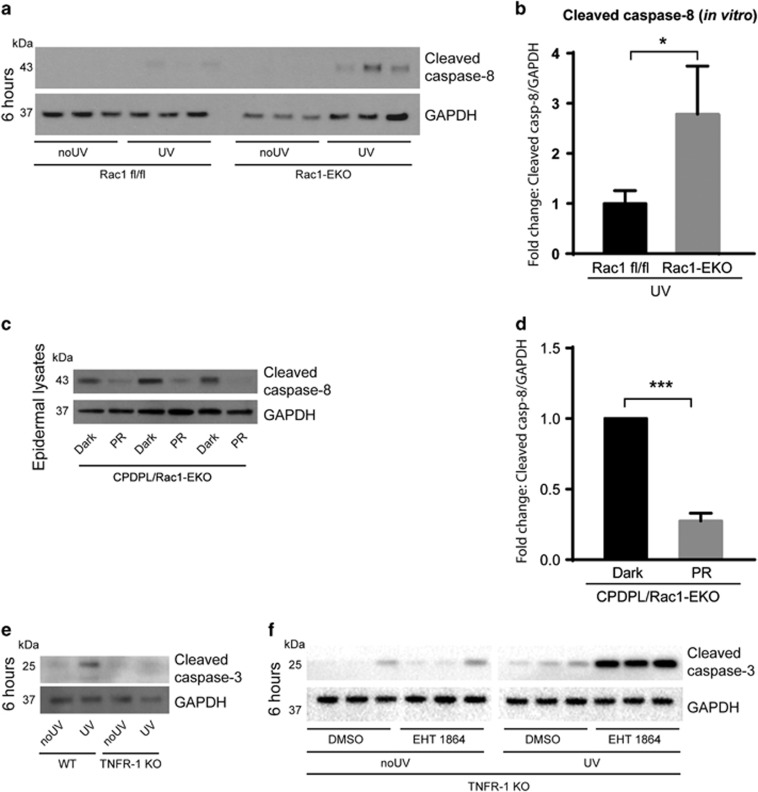
Increase in UV-light-induced apoptosis in Rac1-deficient keratinocytes requires activation of caspase-8. (**a**) Western blot analysis of cleaved caspase-8 from Rac1 fl/fl and Rac1-EKO cultured keratinocytes at 6 h with (UV) or without (no UV) UV-irradiation. (**b**) Densitometry analysis of fold change of cleaved caspase-8 in (**a**) normalized to GAPDH in Rac1 fl/fl and Rac1-EKO samples after UV-irradiation. (**c**) Western blot analysis of cleaved caspase-8 from CPDPL/Rac1-EKO epidermal lysates from mice kept in the dark or under the photoreactivtion lamp (PR). (**d**) Densitometric analysis of cleaved caspase-8 western blots in **c**. Error bars show S.D. (**e**) Western blot analysis of cleaved caspase-3 from wild-type (WT) and TNF receptor-1-deficient (TNFR-1 KO) cultured keratinocytes at 6 h with (UV) or without (no UV) UV-irradiation. (**f**) Western blot analysis of cleaved caspase-3 from TNFR-1 KO cultured keratinocytes incubated with DMSO or Rac1 inhibitor (EHT 1864) at 6 h with (UV) or without (no UV) UV-irradiation. GAPDH is used as a loading control. Numbers on the left denote molecular weights in kDa. * and *** represent *P*-value<0.05 and <0.001, respectively

To investigate the involvement of the intrinsic/extrinsic apoptotic pathways, we analyzed activation of caspase-8 and caspase-9 in Rac1-deficient primary keratinocytes. While we did not detect differences in the cleavage of caspase-9 (data not shown), cleaved caspase-8 was detectable at low levels in Rac1 fl/fl but at much higher levels in Rac1-EKO samples (Figures 7a and b) at 6 h after UV-irradiation. In addition, specific inhibition of caspase-8 with Z-IETD-FMK revealed that the increased apoptosis in Rac1-EKO keratinocytes upon UV-irradiation is dependent on caspase-8 activity (Supplementary Figure S5).

To investigate whether activation of caspase-8 is also dependent on UV-light-induced DNA damage, we irradiated CPDPL/Rac1-EKO mice as described above. Western blot analysis of epidermal lysates for cleaved caspase-8 showed strong bands in the Dark group and much weaker bands in PR group (Figures 7c and d).

These data show that the UV-light dependent, CPD-mediated increase in apoptosis in Rac1-EKO keratinocytes correlates with and depends on activation of caspase-8.

Initiation of the extrinsic, caspase-8-dependent apoptotic pathway occurs after the activation of death receptors such as Fas, TNF receptor 1 and TRAIL receptor2.^18, 19^ To investigate the involvement of TNFR-1, we carried out UV-irradiation experiments on keratinocytes isolated from TNFR-1 knockout (TNFR-1 KO) mice and wild-type mice. In accordance with previous *in vivo* studies^39^ western blot analysis showed strikingly weaker bands for cleaved caspase-3 in TNFR-1 KO keratinocytes than in wild-type keratinocytes after UV-irradiation (Figure 7e), suggesting that UV-light-induced apoptosis in wild-type keratinocytes is majorly TNFR-1 dependent.

To investigate whether also the increase in UV-light-induced apoptosis in Rac1-EKO keratinocytes is TNFR-1 dependent, we pharmacologically inhibited Rac1 in TNFR-1 KO keratinocytes using the specific Rac1 inhibitor EHT-1864. Western blot analysis for cleaved caspase-3 showed weak bands in non-irradiated TNFR-1 KO keratinocytes incubated with DMSO or EHT-1864 (Figure 7f). Much stronger bands for cleaved caspase-3 were observed in TNFR-1 KO keratinocytes incubated with EHT-1864 than in DMSO controls at 6 h after UV-irradiation (Figure 7f). These data suggest that the increase in UV-light-induced apoptosis in Rac1-deficient keratinocytes occurs through TNFR-1 independent mechanisms.

### Epidermis-specific deletion of Rac1 facilitates SCCs upon long-term UV-irradiation

**Figure 8:**
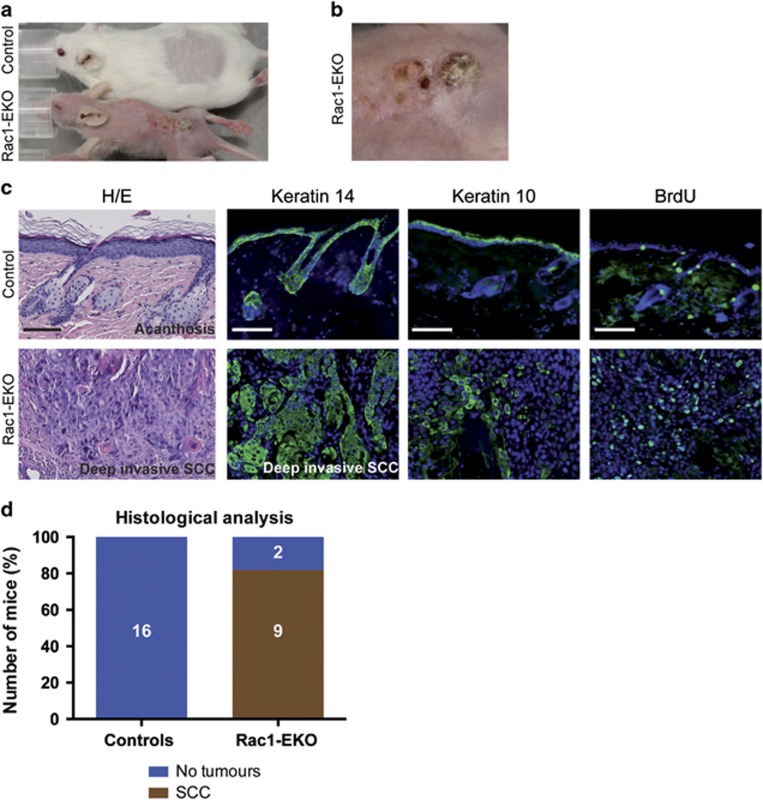
Epidermis-specific deletion of Rac1 facilitates development of SCC upon long-term UV-irradiation. (**a** and **b**) Representative photographs of the mice with hyperkeratotic papules in healed skin erosions of Rac1-EKO mice. The image in **b** is a close-up of the lesion seen in the Rac1-EKO mouse in (**a**). (**c**) H/E staining and immunostainings against keratin 14, keratin 10, and BrdU (green) of skin and tumor samples of controls and Rac1-EKO mice. Nuclei are stained in blue. Scale bar=100 *μ*m. (**b**): Graph shows the results of the histological analysis of skin samples or tumors from control and Rac1-EKO mice. Mice without tumors are shown in blue and mice with SCCs in gray. Absolute numbers of mice are given within the bars

In our long-term UV-irradiation experiments, only Rac1-EKO mice developed skin erosions. When these erosions reached the size of the irradiation field, irradiation had to be stopped. We then followed up 11 of these mice. All of the skin erosions healed within 4-6 weeks without visible scarring. Beginning 4 weeks after the end of the treatment, we noticed the development of small, coalescing, skin colored nodules, which subsequently became covered by scales and crusts in 5 out of 11 (45%) Rac1-EKO mice. No macroscopic skin changes were observed in control mice (Figures 8a and b). Mice were sacrificed and their skin was subjected to investigator blinded histological assessment. Histological analysis of skin from control mice revealed slight acanthosis without any other significant changes (Figure 8c). In contrast, microscopic analysis of the skin samples from the healed skin erosions of Rac1-EKO mice revealed clear histological signs of malignancy in samples from 9 out of 11 (81.8%) mice. In these samples, we observed asymmetric and invasive growth, cellular and nuclear pleomorphism, disturbed differentiation, and an increased mitotic rate (Figure 8c). These histological features were only observed in Rac1-EKO mice, but not in control mice.

To further analyze these tumors, we carried out immunostainings. In control mice, Keratin 14 immunostaining showed keratin 14-positive cells confined to the basal layer of the epidermis with clear demarcation of dermis and epidermis, whereas in Rac1-EKO mice keratin 14 was expressed throughout the tumor and showed no clear demarcation of dermis and epidermis in certain areas (Figure 8c). In Rac1-EKO mice, immunostaining against the differentiation marker keratin 10 was reduced within the tumors, whereas in control mice keratin 10 staining was regular and confined to the suprabasal layers (Figure 8c). Stainings for incorporated BrdU was increased in the tumors of Rac1-EKO mice compared with control skin (Figure 8c). Therefore, the tumors in Rac1-EKO mice were classified as SCCs. All SCCs in Rac1-EKO mice showed a deep penetrating growth pattern (Figure 8c). These data show that the absence of Rac1 facilitates malignant skin tumor development upon chronic UV-irradiation in mice. Hence, epidermal Rac1 protects from the development of UV-light-induced SCC.

## Discussion

### Epidermal Rac1 protects from UV-light-induced keratinocyte apoptosis

In several experimental settings and in various cell types, Rac1 has been shown to either promote or inhibit apoptosis.^21, 22, 23, 24^ Keratinocytes deficient for the Rac1 activator Tiam1 show increased apoptosis upon growth factor deprivation or heat shock treatment, supporting an anti-apoptotic role of Rac1 in keratinocytes.^20^ Our results provide direct evidence for an important function of Rac1 in the inhibition of UV-light-induced apoptosis in epidermal keratinocytes. Increased keratinocyte apoptosis provides a plausible explanation for the observed formation of skin erosions in Rac1-EKO mice. Other possible mechanisms, such as mechanical irritation of the skin or epidermal necrosis are not likely to have a role since the erosive lesions at the back were inaccessible to scratching and the UV-light doses used for the chronic irradiation experiments were sub-erythemal and, therefore, could not cause skin necrosis.

In addition to skin erosions in chronic UV-irradiation experiments, we consistently observed detachment of the epidermis from the underlying dermis in Rac1-EKO mice but not in their controls 24 h after a single dose of UV-irradiation (Supplementary Figure S1D). It is known that massive keratinocyte apoptosis as a result of severe allergic drug reactions causes a similar epidermal detachment, for example, in Stevens-Johnson syndrome or Toxic epidermal necrolysis.^40, 41^ Hence, it is well conceivable that the increased UV-light-induced keratinocyte apoptosis in Rac1-EKO mice is the cause for both the epidermal detachment and the skin-erosions observed in long-term UV-irradiation studies.

### DNA damage has an important role in increased UV-light-induced keratinocyte apoptosis in Rac1-EKO mice

UV-light-induced DNA damage such as CPDs are a major cause for keratinocyte apoptosis *in vivo*.^38^ Our *in vivo* studies in CPDPL/Rac1-EKO mice suggest that the increase in UV-light-induced apoptosis in Rac1-EKO mice is DNA damage dependent. Although the reduction in the number of CPD-positive cells correlates well with the decrease in the number of sunburn cells and cleaved caspase-3-positive cells, it is possible that other, DNA damage independent mechanisms contribute to increased keratinocyte apoptosis in Rac1-EKO mice.

### Altered DDR upon UV-irradiation in Rac1-deficient keratinocytes

Rac1 has been shown to have an important role in the regulation of DDR and repair pathways.^25, 26^ In accordance with these studies our *in vitro* experiments demonstrate that deletion of Rac1 reduces the UV-light-induced DDR as shown by reduced levels of *γ*-H2AX, p53, and pChk1 in primary mouse keratinocytes (Figure 6). These data suggest a function for Rac1 in the regulation of the UV-light-induced DDR in epidermal keratinocytes. Interestingly, Rac1-EKO keratinocytes failed to up regulate p53 upon UV-irradiation, thus making it conceivable that the increased apoptotic response was not exclusively dependent on the intrinsic pro-apoptotic pathway.

### Involvement of the extrinsic pro-apoptotic pathway in UV-light-induced apoptosis

The extrinsic pro-apoptotic pathway was activated in Rac1 fl/fl and Rac1-EKO keratinocytes upon UV-irradiation, as shown by cleavage of caspase-8 (Figures 7a and b). Furthermore, in accordance with previous studies in TNFR-1 KO mice,^39^ our *in vitro* studies with caspase-8 inhibitor showed that this pathway was required for UV-light-induced apoptosis in Rac1-EKO keratinocytes (Supplementary Figure S5). Sensitization of TNFR-1-deficient keratinocytes to UV-light-induced apoptosis by pharmacological inhibition of Rac1 suggests that the caspase-8-dependent mechanism leading to an increase in UV-light-induced apoptosis is independent from TNFR-1. Thus, increased UV-light-induced apoptosis in Rac1-EKO keratinocytes involves and requires activity of the extrinsic, caspase-8-dependent pathway, but not TNFR-1. The correlation between cellular CPD load and caspase-8 activity (Figure 7c) suggests an overlap between intrinsic and extrinsic UV-light-induced pro-apoptotic pathways in epidermal keratinocytes. The possible involvement of other death receptors such as Fas or TRAIL-R2 remains open at the moment.

### Epidermal Rac1 protects from UV-light-induced SCCs

Epidermis-specific deletion of Rac1 led to the development of SCCs in our chronic UV-irradiation experiments (Figure 8). This is particularly surprising, since Rac1 has been reported to be essential for skin papilloma formation in chemical carcinogenesis experiments.^32^ On the other hand, deficiency of the Rac1 activator Tiam1 inhibited skin papilloma formation but facilitated the development of SCCs in mouse skin, which corresponds to our findings. In previous studies with FVB/N wild-type mice in a similar UV-irradiation treatment regimen, first skin tumors appeared after 248 days of irradiation on average.^37^ In our long-term UV-irradiation experiments, however, we were forced to stop UV-irradiation after 134 days because by then the Rac1-EKO mice developed severe skin erosions. The much lower UV-light dose received in our long-term UV-irradiation experiments provides a plausible explanation for the fact that mice in the control group did not develop any skin tumors. Although the exact molecular mechanisms are not entirely clear, our data make it conceivable that protection from both apoptosis and SCC development by Rac1 could be due to a common mechanism: the regulation of the DDR. When Rac1 is absent from epidermal keratinocytes, the normal DDR to UV-light is disturbed, which results in increased apoptosis. Cells, which escape apoptosis, would accumulate unrepaired DNA damage and, therefore, could serve as the origin for the SCCs, which we observed in our long-term UV-irradiation experiments.

Although inhibition of the DDR appears to be the most likely mechanism to explain the development of SCCs in Rac1-EKO mice, we cannot exclude other hypothetical mechanisms, for example, disturbance of cell polarity and adhesion or modulation of epidermal stem cell properties leading to increased UV light-induced mutagenesis.^42, 43, 44, 45, 46, 47^

Chronic inflammation has been proposed to support the development of cancer.^48, 49^ In accordance with a previous study,^50^ our *in vivo* data show an increased inflammatory response in Rac1-EKO mice upon UV-irradiation as shown by increased numbers of *γδ* T cells and neutrophils in the skin of Rac1-EKO mice (Supplementary Figure S4). Hence, an increased inflammatory response upon UV-irradiation in Rac1-EKO mice could contribute to the development of skin SCCs.

Our results provide, for the first time, evidence for a tumor suppressing function of Rac1 in the epidermis. Regulation of UV-light-induced DDR by Rac1 may be connected to its tumor suppressive function in the epidermis. This function must be taken into account by strategies in which Rac1 is used as a therapeutic target in skin.

## Materials and Methods

### *In vivo* experiments

All animal experiments were conducted in accordance with European, national and institutional guidelines and were approved by local governmental authorities. For single-dose UV-irradiation experiments, mice in a pure C57/Bl6 background were used and for chronic UV-irradiation experiments, mice in a pure FVB/N background were used. Experimental groups of mice were matched for age and sex.

### Chronic UV-irradiation experiments

Rac1-EKO mice^34, 36^ and their controls (Rac1 fl/fl, Rac1 fl/+ and K14Cre Rac1 fl/+) were irradiated repeatedly with UVB light using a protocol described previously.^37^ Briefly, mice were irradiated three times per week with a gradually increasing dose under anesthesia with 2.5% Isofluoran. Mice were shaved before the start of the experiment and at regular intervals thereafter. Mice received a UVB dose of 0.23 J/cm^2^ for the first 12 treatments, 0.41 J/cm^2^ for treatments 13-36, 0.51 J/cm^2^ for treatments 37–48 and 0.61 J/cm^2^ from treatment 49 till the end of the experiment. On average, each mouse received a cumulative dose of >20 J/cm^2^ UVB.

Mice were monitored during each treatment for their general condition, changes in skin morphology and tumor formation. Time to tumor development was taken until a visible nodule in the irradiation field appeared. The UV-irradiation treatment was stopped for mice with severe skin erosions and their controls. Mice were photographed typically once in a week to monitor the skin changes.

### Single-dose UV-irradiation experiments

Back skin of the mice was shaved using an electric clipper one day before UV-irradiation. Mice were anesthetized with intraperitonial injection of 200 *μ*l anesthetic solution Ketavet (Pharmacia, New Jersey, NJ, USA) 10 mg/ml (500 *μ*l), Rompun (Bayer, Leverkusen, Germany) 0,1% (250 *μ*l), NaCl 0,9% (4.25 ml) and Bepanthen eye cream (Bayer) was applied to the eyes shortly before UV-irradiation. The back skin of each mouse (area of ~4 cm^2^) was irradiated while the rest of the skin was covered with UV-light impermeable aluminum foil. The non-irradiated area of back skin was used as an internal control (no UV control). The irradiation experiments were performed with a broadband UV lamp (Teilkoerper UV Therapiesystem TP-4 equipped with Waldmann UV-6 UVB bulbs, Herbert Waldmann GmbH & Co. KG, Villingen- Schwenningen). Mice were irradiated with a single dose of UVB (1 J/cm^2^) and were killed to collect skin samples for further analysis after 6 h, 12 h, or 24 h.

### Photoreactivation experiments

Mice expressing a CPD photolyase (CPDPL) transgene driven by a keratin 14 promoter^38^ were kindly provided by Prof Jan Hoeijmakers. These CPDPL mice were bred with Rac1-EKO mice to obtain CPDPL/Rac1-EKO mice. CPDPL/Rac1-EKO mice were irradiated with a single-dose of UV-light as described above. One group of mice was immediately kept in the dark and another group was placed under the photoreactivation lamp made up of 5 Philips TL-D 15 W/33-640 tubes (Philips, Amsterdam, Netherlands). After 12 h mice were killed and the skin samples were collected for further analysis.

### BrdU injection of mice

To analyze proliferation, mice were injected with 200 *μ*l of 16 mg/ml BrdU (Serva, Heidelberg, Germany) (No. 15240) per adult mouse 1 h before the mice were killed and tissue samples were collected.

### Separation of epidermis and dermis

Mice were killed and whole-skin tissue was isolated. Mouse epidermis was scraped off after incubating whole-skin tissue samples in 0.5 M ammonium thiocyanate (NH_4_SCN) in phosphate buffer, pH 6.8 (0.1 M NH2HPO4, 0.1 M KH2PO4) for ~30 min on ice and was snap frozen in liquid nitrogen immediately.

### Histopathology and immunostaining

Tumors and skin samples were excised and tissues were fixed in 4% formalin and subsequently embedded in paraffin. H/E stained tissue sections were obtained using standard procedures. Histopathological examination and immunostainings were carried out as described previously.^35^ Primary antibodies against cleaved caspase-3 (AF835) (R and D systems, Minneapolis, MN, USA), CPD (Thymine dimers, MC-068) (Kamiya biomedicals, Seattle, WA, USA), keratin 14 (PRB-155-P) (Covance, Princeton, NJ, USA), keratin 10 (PRB-159-P) (Covance) and BrdU (347580) (BD biosciences, San Jose, CA, USA) were used. Alexa 488 linked anti-rabbit (A11034) and anti-mouse (A21121) (Invitrogen, Carlsbad, CA, USA) secondary antibodies were used.

### Primary mouse keratinocyte isolation and culture

Primary epidermal keratinocytes from new-born FVB/N mice at post-natal day 0–3 (P0-P3) were isolated. Briefly, mice were decapitated, decontaminated by serial washes in povidone-iodine solution, sterile PBS, 70% EtOH and antibiotic-antimycotic solution (Gibco, Life Technologies, Carlsbad, CA, USA) (No. 15240-096) for 30 s each. Whole-skin samples were collected from the torso and incubated with 5 mg/ml dispase II (Sigma-Aldrich, St. Louis, MO, USA) (No. D4693-1G) in keratinocyte culture medium overnight at 4 °C. The epidermis was separated and was incubated with TrypLE (Gibco) (No. 12604-013) for 30 min at RT. Afterwards, basal keratinocytes were isolated from the epidermis by several washes with low calcium FAD medium,^51^ and cultured on collagen type-1 coated cell culture plates in a cell culture incubator at 34 °C and 5% CO_2_.

### UV-irradiation experiments *in vitro*

Keratinocyte culture medium was removed from cultured keratinocytes and keratinocytes were washed once with sterile PBS. Cells were irradiated with 0.5 J/cm^2^ dose of UV-light while in the thin PBS layer covering the cells. After UV-irradiation, PBS was removed and cells were incubated in culture medium for the indicated time points.

### CPD ELISA

Keratinocytes in culture were irradiated with 50 mJ/cm^2^ as described above and were harvested immediately. Genomic DNA was purified using the isopropanol precipitation procedure. CPDs were quantified by ELISA with monoclonal antibodies (TDM-2 Kamiya biomedicals, USA) (No: MC-062). The protocol was adapted from Nakagawa *et al.*, 1998.^52^ In brief, 96-well plates (VWR, USA) (No. 62409-024), were coated with sample DNA (200 ng per well in triplets). Binding of CPD-specific monoclonal antibodies to immobilized DNA was detected with HRP-labeled goat anti-mouse antibody (Cell Signaling Technology, Danvers, MA, USA) (7076) using TMB substrate (Thermo Fisher Scientific, Waltham, MA, USA) (No. PI34028). The absorbance of colored products was measured at 450 nm with a reference of 620 nm using a plate reader (Perkin Elmer, Waltham, MA, USA).

### Inhibition studies

Keratinocytes in culture were incubated with or without the caspase-8- [Z-IETD-FMK (FMK 007)] specific inhibitor (R&D systems, USA) from 1 h before the UV-irradiation until they were harvested.

### Western blotting

Skin tissue or cultured keratinocytes were lysed with lysis buffer (1% SDS, 10 mM EDTA). Extracted proteins were denatured at 100 °C for 5 min and subjected to electrophoresis using 4-12% Bis-Tris gels (Novex, Life Technologies, Carlsbad, CA, USA) (No. BG04125BOX). Gels were blotted onto polyvinyldiene fluoride membranes using the iBlot transfer system (Invitrogen, Life Technologies, Carlsbad, CA, USA). Afterwards, membranes were incubated in blocking solution (Roche Applied Science, Indianapolis, IN, USA) (No. 11921673001) 1:10 in Tris buffered saline with 0.01% Tween 20 (TBST).

Blots were probed with primary antibodies against cleaved caspase-3 1:1000 (R and D systems, USA) (MAB835), p-Chk1 (2348), Chk1 (2360), p53 (2524), *γ*-H2AX (9718), cleaved caspase-9 (9509), cleaved caspase-8 (9429) 1:1000 (Cell Signaling Technology, USA) and GAPDH 1:5000 in TBST (Trevigen, Gaithersburg, MD, USA) (No. 2275). HRP linked anti-rabbit (7074) or anti-mouse (7076) secondary antibodies (Cell Signaling Technology, USA) 1:2000 were used. Immunoreactive proteins were detected using chemiluminescence (PerkinElmer, Waltham, MA, USA) (No. NEL103001EA) and the blots were developed using X-ray films (Thermo scientific, Life Technologies, Carlsbad, CA, USA) (No. 34089).

### Colony forming assays

Typically, low passage primary mouse keratinocytes were plated in 6-well plates (5000 cells per well) and cultured for approximately 2–3 weeks in the presence of mitomycin treated feeder fibroblasts. Fibroblasts were changed once a week. Finally, fibroblasts were removed and keratinocytes were fixed with 1% PFA for 15 min. Subsequently colonies were stained for minimum 1 h with 0.05% crystal violet in PBS. Afterwards, cell culture dishes were washed with dH2O and left for drying 30 min RT. Digital images were obtained using canon camera EOS 1100 D (Canon, Tokyo, Japan).

### Adhesion assays

Primary mouse keratinocytes in passage 1 were seeded on to 96 well plate in triplicates and were incubated for 3 h at 37 °C. After a PBS wash, cells were incubated with lysis buffer (9% TritonX100) for 45 min at 37 °C. Ninety-six-well plates were centrifuged, and 50 *μ*l supernatant from each well was transferred to a fresh 96-well enzymatic assay plate. 50 *μ*l of reconstituted substrate mix from CytoTox 96 Non-Radioactive Cytotoxicity Assay (Promega, Madison, WI, USA) (No. G1780) was added to each well and incubated at room temperature for 30 min protected from light. Fifty microliters of stop solution was added and the absorbance was recorded at 490 nm using TriStar LB941 plate reader (Berthold technologies, Zug, Switzerland). Experimental samples were compared for absorbance and the numbers of cells attached were calculated according to the manufacturer's instructions (G1780, Promega).

### Statistical analysis

Significance between the samples was calculated using the Student's *t*-test using Prism 6 (GraphPad) unless stated otherwise. The asterisks shown in graphs correspond to the *P*-values as stated in the figure legends and text. *P*-value <0.05 was considered as statistically significant. *P*-value <0.05 (*), *P*-value <0.01 (**), *P*-value <0.001 (***).
