# Supplementary figures and images for: Epidermal Rac1 regulates the DNA damage response and protects from UV-light-induced keratinocyte apoptosis and skin carcinogenesis
Source: Cell Death Dis. 2017 Mar 9;8(3):e2664–. doi: 10.1038/cddis.2017.63 (PMC5386559; doi:10.1038/cddis.2017.63)

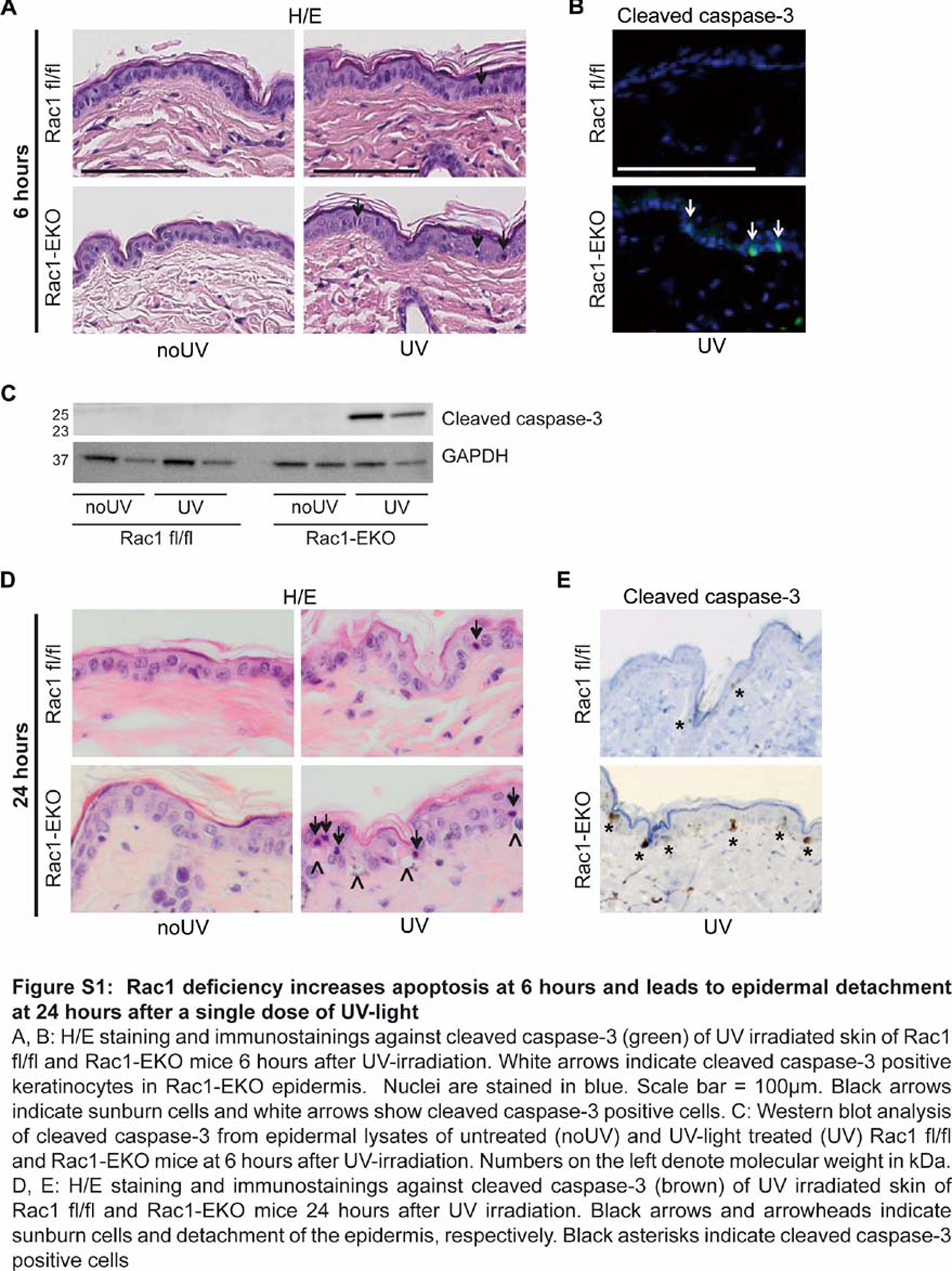

Supplement: Supplementary Figure 1 [file cddis201763x1.tif]

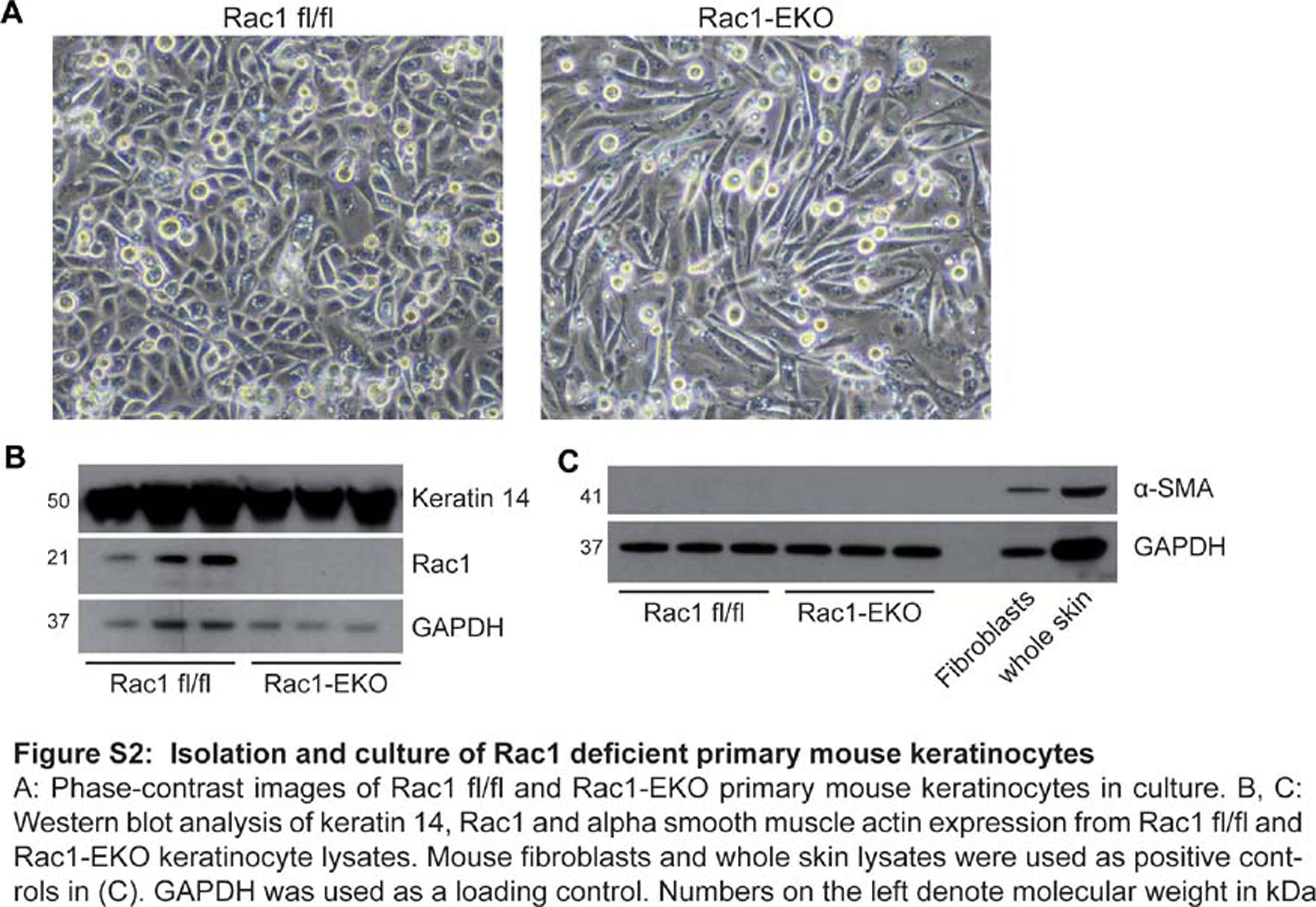

Supplement: Supplementary Figure 2 [file cddis201763x2.tif]

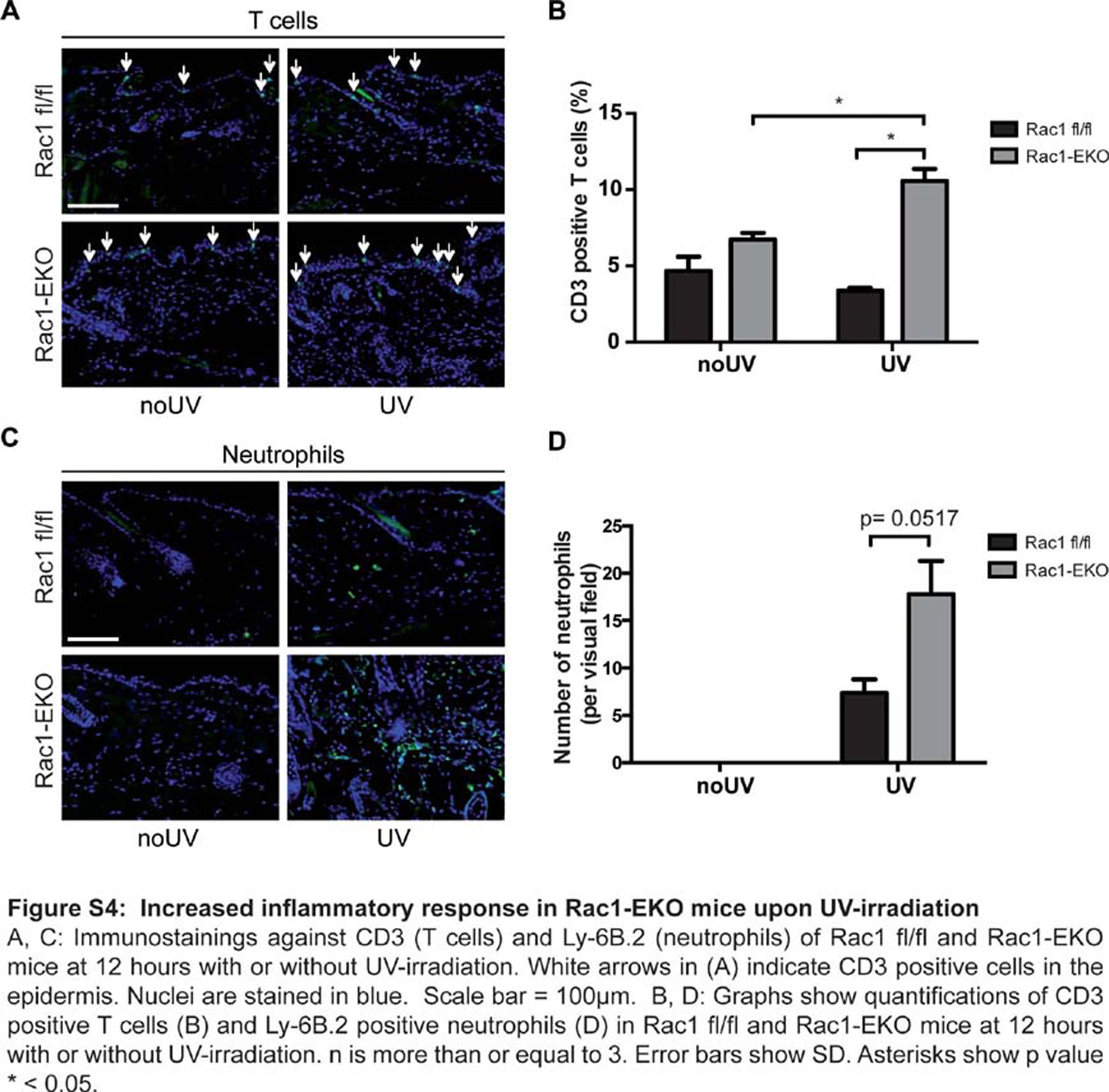

Supplement: Supplementary Figure 4 [file cddis201763x3.tif]

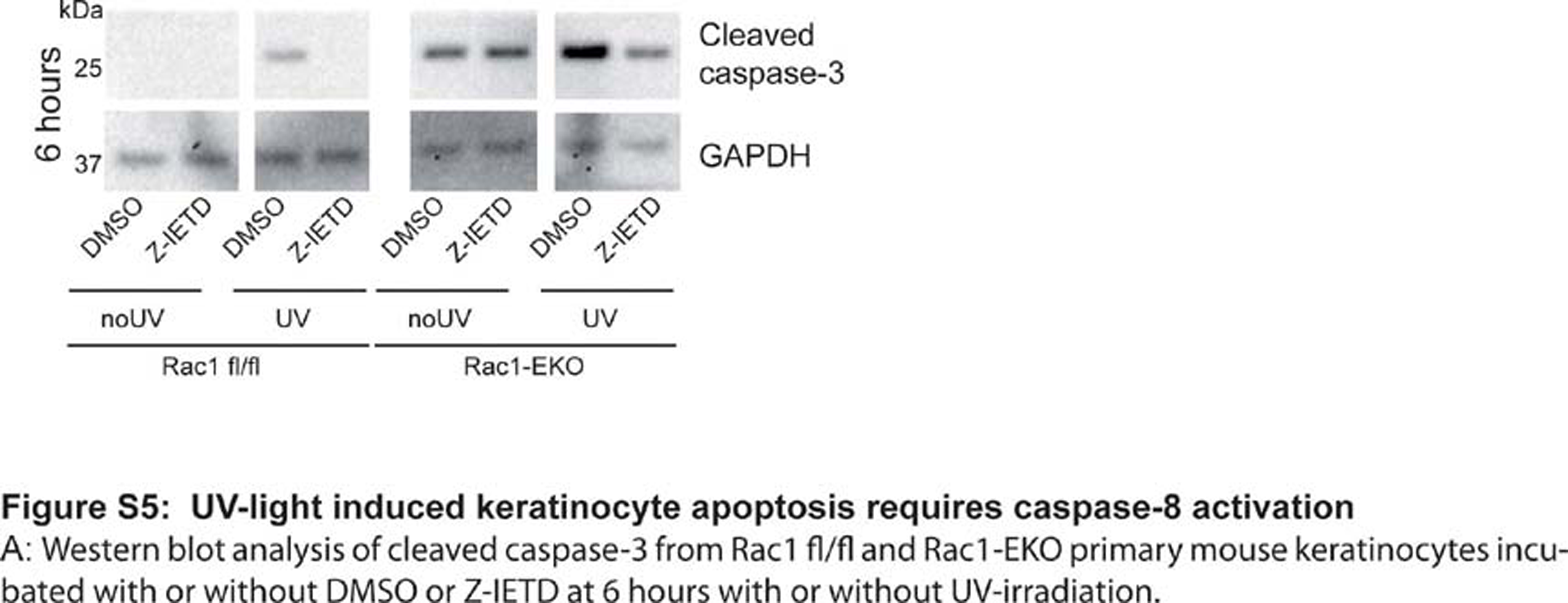

Supplement: Supplementary Figure 5 [file cddis201763x4.tif]
